# Supplementary material for: Pathological Complete Response Following Neoadjuvant Tislelizumab Monotherapy in Treatment-Naive Locally Advanced, MMR-Deficient/MSI-High Ascending Colon Cancer: A Case Report
Source: J Clin Med. 2022 Dec 28;12(1):240. doi: 10.3390/jcm12010240 (PMC9821431; doi:10.3390/jcm12010240)
Supplement: Supplementary file 1 [file jcm-12-00240-s001.zip › Supplementary Table S1. CARE checklist.pdf]

Reporting checklist for case report or case series.

Based on the CARE guidelines.

		Reporting Item	Page Number
**Title**			
	#1	The area of focus and “case report” should appear in the title	1
**Keywords**			
	#2	Two to five key words that identify topics in this case report	2
**Abstract**			
Introduction	#3a	What is unique and why is it important?	2
	#3b	The patient’s main concerns and important clinical findings.	2
	#3c	The main diagnoses, interventions, and outcomes.	2
Conclusion	#3d	What are one or more “take-away” lessons?	2
**Introduction**			
	#4	Briefly summarize why this case is unique with medical literature references.	3
**Patient information**			
	#5a	De-identified demographic and other patient information.	4
	#5b	Main concerns and symptoms of the patient.	4

	#5c	Medical, family, and psychosocial history including genetic information.	4
	#5d	Relevant past interventions and their outcomes.	4
**Clinical findings**			
	#6	Relevant physical examination (PE) and other clinical findings.	4
**Timeline**			
	#7	Relevant data from this episode of care organized as a timeline (figure or table).	No (short course of treatment (neoadjuvant immunotherapy) and the episode is obvious in this case)
**Diagnostic assessment**			
	#8a	Diagnostic methods (PE, laboratory testing, imaging, surveys).	5
	#8b	Diagnostic challenges.	5
	#8c	Diagnostic reasoning including differential diagnosis	No (the pathology of biopsy has been confirmed)
	#8d	Prognostic characteristics when applicable	6 and Supple.
**Therapeutic Intervention**			
	#9a	Types of intervention (pharmacologic, surgical, preventive).	5 and 6
	#9b	Administration of intervention (dosage, strength, duration)	5 and 6
	#9c	Changes in the interventions with	5 and 6

		explanations.	
**Follow up and outcomes**			
	#10a	Clinician and patient-assessed outcomes when appropriate	6
	#10b	Important follow-up diagnostic and other test results.	6
	#10c	Intervention adherence and tolerability (how was this assessed)?	6
	#10d	Adverse and unanticipated events.	6
**Discussion**			
	#11a	Strengths and limitations in your approach to this case.	8
	#11b	Discussion of the relevant medical literature.	7and 8
	#11c	The rationale for your conclusions.	8
	#11d	The primary “take-away” lessons from this case report.	8 and 9
**Patient perspective**			
	#12	The patient can share their perspective on their case	No (The patient signed the informed consent form and had no comments on the paper)
**Informed consent**			
	#13	The patient should give informed consent.	yes

None The CARE checklist is distributed under the terms of the Creative Commons Attribution License CC-BY-NC. This checklist can be completed online using https://www.goodreports.org/, a tool made by the EQUATOR Network in collaboration with Penelope.ai
